# Success and Controversy of Natural Products as Therapeutic Modulators of Wnt Signaling and Its Interplay with Oxidative Stress: Comprehensive Review Across Compound Classes and Experimental Systems

**DOI:** 10.3390/antiox14050591

**Published:** 2025-05-14

**Authors:** Alexey Koval, Nilufar Z. Mamadalieva, Rano Mamadalieva, Fazliddin Jalilov, Vladimir L. Katanaev

**Affiliations:** 1 Translational Research Center in Oncohaematology, Department of Cell Physiology and Metabolism, Faculty of Medicine, University of Geneva, 1211 Geneva, Switzerland; n.mamadaliyeva@afu.uz; 2 Faculty of Medicine, Alfraganus University, Yuqori Qoraqamish Str. 2a, Tashkent 100190, Uzbekistan; f.jalilov@afu.uz; 3 Institute of the Chemistry of Plant Substances, Uzbekistan Academy of Sciences, Mirzo Ulugbek Str. 77, Tashkent 100170, Uzbekistan; 4 Faculty of Medicine, Namangan University of Business and Science, Beshkapa Str. 111, Namangan 160100, Uzbekistan; r.mamadaliyeva@ubsu.uz; 5 Translational Oncology Research Center, Qatar Biomedical Research Institute (QBRI), College of Health and Life Sciences, Hamad Bin Khalifa University (HBKU), Qatar Foundation, Doha P.O. Box 34110, Qatar

**Keywords:** Wnt signaling, natural compounds, antioxidant, preclinical models, screening

## Abstract

The highly conserved Wnt signaling pathway, a complex network critical for embryonic development and adult tissue homeostasis, regulates diverse cellular processes, ultimately influencing tissue organization and organogenesis; its dysregulation is implicated in numerous diseases, and it is known to be affected by oxidative pathways. This report reviews the recent literature on major classes of natural products with pronounced anti-oxidant properties, such as cardiac glycosides, steroid saponins, ecdysteroids, withanolides, cucurbitacins, triterpenes, flavonoids, and iridoids, that modulate its activity in various pathological conditions, summarizing and critically analyzing their effects on the Wnt pathway in various therapeutically relevant experimental models and highlighting the role of ROS-mediated crosstalk with Wnt signaling in these studies. Models reviewed include not only cancer but also stroke, ischemia, bone or kidney diseases, and regenerative medicine, such as re-epithelialization, cardiac maintenance, and hair loss. It highlights the paramount importance of modulating this signaling by natural products to define future research directions. We also discuss controversies identified in the mode of action of several compounds in different models and directions on how to further improve the quality and depth of such studies.

## 1. Introduction

The Wnt signaling pathway is a complex and broadly defined intracellular signaling network that plays a key role in embryonic development and adult tissue homeostasis. It is involved in a wide range of processes at the cellular level, including cell fate determination, cell migration, cell polarity, and ultimately the control of tissue organization and organogenesis. The pathway is highly conserved across species and, when dysregulated, is implicated in numerous human diseases and pathological processes. The natural products in the current review have been used to either modulate pathological involvement or benefit from the activation of this pathway in the following situations:

- Almost all major cancer types, such as esophageal, breast, lung (predominantly non-small-cell lung carcinoma, NSCLC), liver, colorectal, ovarian, prostate cancers, as well as osteosarcoma, leukemia, melanoma, and glioma [1];
- In a range of renal pathologies, such as renal epithelial cell toxicity [2], chronic kidney disease [3,4], and renal fibrosis [5];
- Plays an important role in bone homeostasis, development and pathology by regulating osteoblast differentiation [6,7,8,9], with particular implications for arthritis [10,11,12] and obesity-induced bone loss [13];
- Neuronal tissue function, with roles in such devastating conditions as stroke [14,15,16], spinal cord injury [17], and demyelination [18];
- Significant roles in several intestinal ailments, such as intestinal ischemia–reperfusion injury [19] or intestinal dysfunction induced by peritoneal dialysis [20];
- Involved in cardiac maintenance and recovery, particularly cardiac hypertrophy [21];
- Influences key cell types in the hair follicle to promote hair growth [22];
- Inhibition of the cascade is useful against damage-related lung fibrosis [23] and fibrosis resulting from unilateral ureteral obstruction [24];
- Activation of the cascade impairs re-epithelialization by keratinocytes, impeding wound healing [25];
- Pathway inhibition is useful to protect against the consequences of retinal ischemia [26].

**Figure 1:**
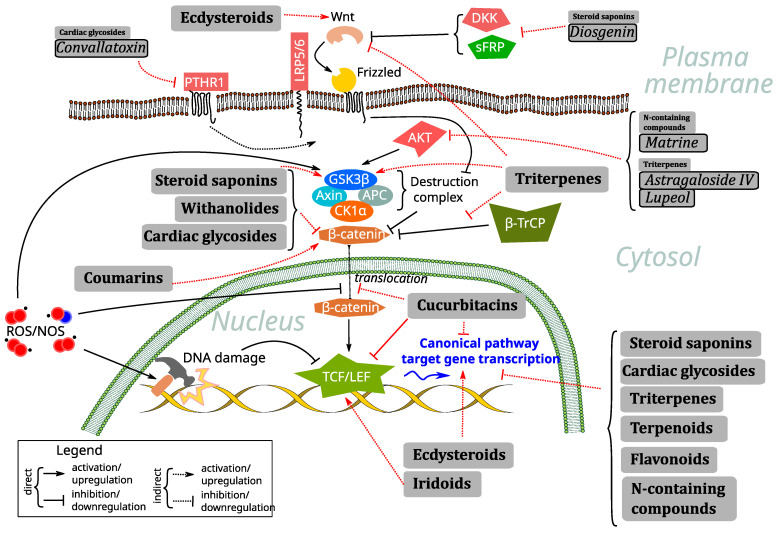
Principal scheme of the Wnt pathway along with its crosstalk with RO/NS and representation of the main effects of the classes of compounds reviewed in the current study, along with several representatives of these classes with activities unusual for the class.

The Wnt signaling pathway (Figure 1) can be broadly divided into the canonical (β-catenin-dependent) and non-canonical (β-catenin-independent) pathways. The canonical pathway is activated when Wnt proteins bind to Frizzled (FZD) receptors and LRP5/6 co-receptors. At this level of membrane signaling, they can be antagonized by natural secreted antagonists of the DKK family (e.g., DKK1), which bind to LRP5/6 and prevent interaction with Wnt; the sFRP family (e.g., sFRP4), which antagonize Wnt binding to FZD; or WIF1, which binds to Wnt ligands themselves and prevents their function. Downstream transduction of the signal occurs upon hyperphosphorylation of LRP and, with the help of Dishevelled, a scaffold protein downstream of FZD, leads to disruption of the β-catenin destruction complex, which consists of 2 scaffold proteins, Axin and APC, and 2 kinases, glycogen synthase kinase 3β (GSK3β) and casein kinase 1 alpha (CK1a). In the absence of a membrane-emerging signal, the destruction complex is constitutively active; it catalyzes CK1a, which primes beta-catenin phosphorylation at Ser45, and promotes GSK3β to complete this process at Ser33/37/Thr41. However, the activity of GSK3β in this process can be tuned by its own phosphorylation at Ser9 (inhibitory, typically made by AKT kinase) and Tyr216 (activating, typically autophosphorylation). In the presence of a signal from the Wnt/FZD interaction, the destruction complex dissociates and ceases to function, leading to the cytoplasmic accumulation and subsequent nuclear translocation of β-catenin. This process, both in terms of improved protein stability and enhanced translocation, is stimulated by Ser552/675 phosphorylation of β-catenin by various kinases, including AKT, and by the nuclear transport protein galectin-3. In the nucleus, β-catenin associates with homologues of the TCF/LEF transcription factors (including TCF1, TCF7, TCF4, and LEF1), to regulate the expression of a variety of target genes. They are involved in a wide variety of processes, such as general cell cycle control (c-Myc, cell-cycle-related kinase CCRK, and cyclin D1), anti-apoptotic function (survivin), RNA regulation (CRD-BP), a variety of transcription factors controlling cell fate and differentiation (Runx2, Twist, Id2, MITF, Sox9), extracellular matrix remodeling (MMP1, 2, 7, and 9, PAI-1), xenobiotic efflux pumps (MDR1, ABCC1), regulation of the activity of other signaling pathways (Jagged), and even Wnt signaling proper negative feedback (Axin2). Pathway activity is often assessed using TopFlash, a luciferase-based reporter system driven by the TCF family of transcription factors. There is also a significant repertoire of small-molecule inhibitors of the Wnt pathway available for research purposes, such as the tankyrase inhibitors WIKI4 and XAV939, which inhibit the pathway by preventing Axin degradation, or KYA1797K, which inhibits the pathway by potentiating Axin interaction with β-catenin. On the other hand, the pathway can be activated by direct inhibition of GSK3β using small molecules such as SB-216763.

Agents targeting canonical Wnt signaling can be divided into two major subtypes: small-molecule inhibitors and monoclonal antibodies. The former usually refer to chemical compounds with a molecular weight of less than 1 kDa [27]—a definition which perfectly fits the majority of natural and nature-derived compounds. Among them, triterpenes [28], withanolides [29], steroidal saponins [30], taninns [31], proanthocyanidins [32], diterpenes [33,34,35], alkaloids [36,37,38], lignans, sesquiterpene lactones, sterols, and polysaccharides [39] have been reported as plant-based inhibitors of Wnt signaling. Their mechanism of action frequently involves the regulation of ROS/RNS (reactive oxygen species/reactive nitrogen species)-mediated crosstalk with the Wnt pathway (Figure 1).

ROS/RNS, also known collectively as RO/NS, have long been considered to be generally detrimental byproducts that cause varying degrees of oxidative stress—a condition with diverse effects, mostly related to quantitative overload [40]. This group of effects can be summarized in two main mechanisms:

- **Effects on DNA and protein integrity:** RO/NS are capable of directly damaging DNA bases, causing strand breaks and adducts, which, in turn, may cause mutations that lead to genomic instability and may accelerate the carcinogenesis process [40]. Depending on the exact levels and nature, there could also be quantitative effects on protein integrity and cross-linking. Obviously, this effect is mostly directly counteracted by the scavenging ability of antioxidant compounds [40,41].
- **Impact on ferroptosis:** This type of cell death is characterized by iron-dependent lipid peroxidation. The formation of lipid hydroperoxides initiates the process, which ultimately leads to cell membrane damage and cell death in the presence of iron [42,43]. Fenton-like reactions are then activated to produce lipid radicals [42]. Natural antioxidants can affect ferroptosis in a number of ways, most notably by directly scavenging lipid peroxyl radicals, acting as chain-breaking antioxidants [42], or by altering intracellular GSH levels [43].

Currently, the paradigm of RO/NS understanding is being expanded to also include essential molecules in what is collectively referred to as redox signaling and that are involved in many different physiological processes [44,45]. At low-to-moderate concentrations and with proper localization, RO/NS have been found to function as intracellular messengers [46]. The example of nitric oxide (NO), which is essential for immune defense, neurotransmission, and vasodilation, is well known [47]. However, this one is far from an exception among RO/NS species to control intracellular pathways, as other similar radicals have been found to play a role in intracellular signaling [45]. Events under their control include immunological responses, cell division, and proliferation. For example, localized, brief spikes of superoxide can trigger various transcription factors (NF-κB, NRF2, etc.) and kinases (MAPK, PI3K), which, in turn, mediate specific downstream effects on cells [45].

Therefore, RO/NS can only be considered dangerous at significant excesses, so that by scavenging excess RO/NS with antioxidants, including natural substances, we may end up with “antioxidant overload” instead of rescue. Total suppression of ROS signaling has been shown to disrupt vital functions such as angiogenesis and tissue repair signaling [48] or pathogen defense [49]. Thus, a sophisticated understanding of the ideal RO/NS balance and precision in its application are required. It is also clear that many natural compounds that act not only as antioxidants but also as modulators of various signaling pathways, such as Wnt, additionally contribute to the RO/NS balance through these inputs.

From the vast number of reports included in this review, it is clear that the main source of natural-product-based Wnt inhibitors (as is the case for the activators) has so far been various medicinal plants. For this reason, considering the critical role of β-catenin signaling in cancer development and progression and several targeting strategies, it is crucial to continue the search for a new Wnt inhibitors based on plant compounds.

## 2. Review

### 2.1. Natural Compounds as Mediators and Regulators of Wnt Signaling via Oxidative Stress and ROS Regulation

Wnt signaling has been demonstrated to show an intricate interplay with ROS-mediated pathways and, specifically, in oxidative stress by variety of mechanisms, including directly affecting various component proteins or more generic ones, or via DNA damage repair, and in many different contexts, notably cancer [50] but also in cardiovascular pathology [51] and even in the course of embryonic development [52]. All classes of natural compounds described and evaluated in the current work are known to possess antioxidant activity of different strengths, though in certain contexts, they might also exert surprising pro-oxidant functions, and thus, the observed results are frequently directly derived from exactly this mode of interaction with the Wnt signaling cascade. Surprisingly, as we describe in detail in the following text, several large classes had no reports linking their Wnt-signaling-related activity to their ability to regulate ROS and oxidative stress (cardiac glycosides, withanolides, terpenoids, coumarins). However, a significant overrepresentation of such synergistic effects is seen among saponins, with dioscin, diosgenin, ginsenosides, and timosaponin AIII studied in liver fibrosis, bone-related conditions, vascular calcification, intestinal injury, and in the context of cancer. The ecdysteroid 20-hydroxyecdysone and the triterpene oleanoic acid synergized their antioxidant effects with Wnt pathway activation in neuroprotective functions, while the triterpene astragaloside IV was active against cancer, acting both as an ROS scavenger and pro-oxidant with equally polar effects on Wnt signaling. The iridoid cornin enhanced Wnt expression to promote muscle regeneration along with antioxidant action (see Figure 1 and Table 1 for a summary of the compound activities and mechanisms reviewed).

### 2.2. Cardiac Glycosides

One of the most prominent cardenolides shown to affect Wnt signaling in a variety of contexts is ouabain. The compound is being investigated for its role in reversing cisplatin resistance in the esophageal cancer line EC109. It reduces the translocation of β-catenin to the nucleus, thereby inhibiting the Wnt/β-catenin pathway, as evidenced by a drop in luciferase reporter TopFlash activity, and it contributes to the reversal of resistance by downregulating a Wnt target gene, MDR1 [53]. In two other papers, the results reported the opposite effect of the compound on cells. Thus, in MDCK cells, low concentrations of ouabain can promote the shuttle of β-catenin to the nucleus by increasing its unidentified tyrosine phosphorylation, thus promoting the activation of the signaling pathway [54]. In a different non-cancerous context, the hippocampus of adult rats, the compound increased phosphorylation and, thus, inactivated GSK3β, resulting in decreased phosphorylation and stabilization of β-catenin and its translocation to the nucleus, with pathway activation confirmed by an increase in Axin2 expression, a direct target gene. This activity resulted in increased dendritic arborization and improved spatial reference memory and its morphological plasticity [55].

Other cardenolides have been reported to have exclusively inhibitory effects on Wnt-induced signaling. For example, oleandrin has been reported in at least two articles as a Wnt inhibitor with anti-cancer properties. In the osteosarcoma cell lines U2OS and SaOS-2, it suppresses the TopFlash reporter and target genes (c-Myc, survivin, cyclin D1, MMP-2, MMP-9) at both the mRNA and protein levels. This effect is caused by its ability to inhibit the nuclear translocation of β-catenin and results in anti-tumor effects [56]. In the second study, oleandrin and odoroside A, another similar cardenolide, were found to be effective against breast cancer in both the parental MDA-MB-231 and its radioresistant subclone. Both compounds dose-dependently reduced invasiveness and radioresistance, mainly through suppression of phospho-STAT-3-mediated pathways, with a contribution of Wnt signaling to these effects manifested as a decrease in β-catenin levels [57].

**Figure 2:**
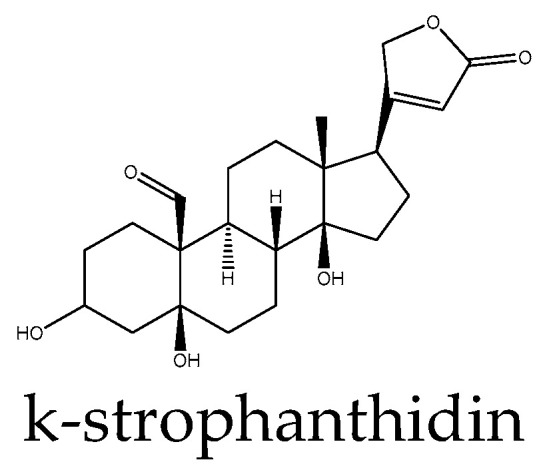
Example structure of a cardiac glycoside, k-strophanthidin, reviewed in the current study.

Peruvoside has been shown to inhibit Wnt signaling in MCF-7 breast cancer, A549 lung cancer, and HepG2 liver cancer cell lines by decreasing β-catenin levels in both the nucleus and cytoplasm, accompanied by a decline in the expression of Wnt-dependent pro-proliferative target genes such as c-Myc and cyclin D1. As a result, it promotes apoptosis via BAX and caspase-3, leading to reduced cancer cell proliferation [58]. The same authors demonstrated the anti-cancer potential of another cardenolide, strophanthidine (Figure 2), in the same cell lines, also via Wnt inhibition. The reported effects on the pathway were essentially the same, with additional effects on PI3K/AKT/mTOR signaling [59]. Nerigoside has been shown to inhibit Wnt signaling by suppressing β-catenin degradation via ERK crosstalk promoting the degradation of GSK3β, thereby exerting an inhibitory effect on colorectal cancer cell lines HT29 and SW620 [60]. Two digitalis-like natural cardenolides, H-9 and ATE-i2-b4, can induce expression of Nur77, an orphan member of the nuclear receptor superfamily, in colorectal cancer cells. Nur77 is exported to the cytoplasm, where it downregulates Wnt signaling through direct interaction and induction of β-catenin degradation via the proteasome, independent of GSK3β and the ubiquitin ligase Siah-1, the latter known to be involved in β-catenin degradation [61]. In the context of osteosarcoma, convallatoxin was found to inhibit the Wnt signaling pathway through crosstalk with parathyroid hormone receptor 1 (PTHR1). Overexpression of PTHR1 or knockdown of the natural inhibitor of Wnt, DKK1, reversed the inhibitory effects of convallatoxin on cancer proliferation, migration, and invasion and enhanced osteogenic differentiation [62]. Finally, calotropin was found to inhibit the Wnt signaling pathway by increasing the levels of casein kinase 1a (CK1a) in colon cancer cells, leading to increased β-catenin degradation through activation of the destruction complex and, ultimately, demonstrating anti-cancer effects [63].

Bufadienolides, closely related to cardenolides, which, in the form of glycosides, are usually referred to together as cardiac glycosides, have also been reported for their activity in Wnt signaling. Similar to strophanthidine above, bufalin inhibits the phosphorylation of AKT, which, in turn, inhibits cell proliferation, migration, and invasion in hepatoma cell lines. AKT activity, in turn, is known to increase the phosphorylation rate of GSK3β, leading to a reduction in its activity towards β-catenin, thereby activating the Wnt signaling pathway [64]. Another study also supports the anti-Wnt capacity of bufalin in liver cancer but by a different mechanism involving cell-cycle-related kinase (CCRK). The compound suppresses CCRK-driven transcription by affecting promoter activity, which, in turn, inactivates Wnt signaling, as evidenced by a loss of active β-catenin, and it thereby suppresses hepatoma line proliferation and tumorigenicity [65]. Telocinobufagin was evaluated outside the cancer context in LLC-PK1 porcine kidney epithelial cell toxicity, and it was found to inhibit Wnt/β-catenin signaling via modulation of GSK3β activity, although AKT involvement was not evaluated. The compound also negatively affected β-catenin stability by a parallel, undefined mechanism, suggesting that it acts at multiple levels in the pathway. Interestingly, the compound was very different in terms of its potency and activity profile from the related marinobufagin, which differs in only one epoxy group [2].

### 2.3. Steroid Saponins

This class of natural compounds was found to inhibit Wnt signaling in most contexts, although there were a few exceptions. Several compounds of this type are reported in only one study, such as ophiopogonin B, which was tested using non-small-cell lung carcinoma cell lines, causing a reduction in β-catenin levels in both the cytoplasm and nucleus, as well as the Wnt target genes cyclin D1 and c-Myc, which, in turn, led to a reduction in cell migration and invasion. Interestingly, the authors observed that Axin was necessary for the inhibition of cell migration by the compound and found that its mechanism of action involved strengthening the Axin/β-catenin interaction [66]. Epibrassinolide reduced lysate β-catenin levels and the expression of pathway target genes such as c-Myc, CCND1, Sox9, c-Jun, Survivin, MMP1, MMP7, and mPar in NCI-H69 and a multi-drug-resistant VPA17 small-cell lung carcinoma line. The authors also compared its activity with another Wnt inhibitor, WIKI4, which acts on tankyrases, showing similar cytotoxicity [67]. Saponin from *Tupistra chinensis* Baker inhibited a pathway in ovarian cancer cells SKOV3 with a hallmark reduction in β-catenin and the pathway downstream target c-Myc. Selectivity was verified using the Wnt activator LiCl, which counteracted the compound’s effects on cell proliferation, apoptosis, and cell cycle [68]. Daucosterol in hepatocellular carcinoma lines HepG2 and SMMC-7721 reduced the levels of β-catenin and Wnt5a while increasing the expression of GSK3β, causing a drop in the proliferation, migration, and invasion capacity of the cells—the effects that were specifically countered by Wnt activation with the GSK3β inhibitor SB-216763 [69].

However, a series of studies are reported for many of these compounds. Paris saponin H was evaluated against hepatocellular carcinoma PLC/PRF/5 and Huh7 cells, where it reduced cell viability and induced apoptosis, including in vivo xenograft, as well as epithelial–mesenchymal transition (EMT) and invasion analyzed only in vitro. It downregulated the expression of β-catenin and levels of p-GSK3β simultaneously, also supported by mimicking compound action by β-catenin silencing [70]. Its close relative, Paris saponin I, was tested in zebrafish, where qPCR experiments measured β-catenin expression, among other genes, and its decrease was reported. The organ toxic effects of the compound in this model were concluded to be mediated, at least in part, by the Wnt pathway [71].

Three studies analyzed Wnt inhibition by polyphyllin I, also called Chong Lou saponin I. As a common feature, in all of them, the authors identified suppression of phosphorylation of GSK3β at Ser9 and a decrease in β-catenin in response to the compound. Exactly this mechanism was associated with in vitro suppression of osteosarcoma cell lines in a panel of in vitro assays and also in a xenograft orthotopic mouse model using intratibial primary tumor [72]. In liver cancer stem cells, this was reported to require AKT crosstalk, with treatment also leading to a loss of β-catenin nuclear accumulation and anti-cancer activity [73]. Outside of cancer, compound administration attenuated cardiac dysfunction, reduced cardiac hypertrophy, and ameliorated cardiac remodeling in a mouse model of pressure-overload-induced cardiac hypertrophy. These effects were mediated by counteracting aberrant activation of Wnt signaling by AngII, as evaluated by analysis of the same Wnt components (β-catenin, p-GSK3β) in model mice and in response to the peptide in vitro in cultured neonatal rat ventricular myocytes, where it also suppressed Wnt target genes c-Myc, c-Jun, c-Fos, and cyclin D1 [21].

At least three studies reported the Wnt inhibitory activity of diosgenin, providing a decrease in β-catenin levels as the main evidence. Thus, it promoted proliferation and differentiation of MG-63 osteoblast-like cells by affecting the expression levels of Wnt target genes Runx2 and cyclin D1—an effect which is also related to its antioxidant action [74]. The effect on HCT-116 human colon carcinoma cells was by the same mechanism, although it should be noted that the authors have shown expression changes in p21ras oncogene and MG-CoA reductase that were also consistent with the observed effects of the compound on cell growth and apoptosis [75]. Finally, in three breast cancer cell lines enriched for cancer stem cells by growing them as mammospheres, the compound acted through upregulation of the Wnt antagonists sFRP4 and DKK1, which made the cells more susceptible to apoptosis caused by specific ROS induction by the compound treatment, as activation of the pathway would help them resist [76].

In human leukemia HL-60 cells, the underlying mechanism of the cytotoxic effect of timosaponin AIII, a proven antioxidant inhibiting proliferation, inducing apoptosis, and causing cell cycle arrest, was linked to the inhibition Wnt pathway. Evidenced by decreased expression levels of β-catenin and target genes cyclin D1 and c-Myc, the authors also found that crosstalk with AKT contributed to its activity in vitro [77]. However, its close analog timosaponin B II in combination with icariin and ferulic acid found in the same extract caused it to reverse its activity. Its effect was studied on UMR-106 osteoblastic cells and primary rat osteoblasts, where it exerted pro-osteogenic effects via increased expression of Dishevelled and an increase in β-catenin levels [7].

Four studies were found implicating dioscin in Wnt signaling. In two of them, the compound induced activation of GSK3β through its decreased phosphorylation at Ser9, promoting β-catenin degradation and downregulating nuclear fraction. The first was carried out in an osteosarcoma model, where dioscin treatment inhibited stem-cell-like properties and xenograft tumor growth. It also blocked AKT activity, and the authors confirmed selectivity to the Wnt pathway by assessing other cancer-related pathways, NICD1 and GLI1, which were not affected, and also by loss-of-compound effects upon β-catenin knockdown [122]. In a second study, dioscin was used in vitro and in vivo in a rat model of oxidative equilibrium shift-based CCl_4_-induced liver fibrosis in primary rat hepatic stellate cells, cultured or isolated from the in vivo study. Inhibition of the pathway was beneficial for the reduction in liver fibrosis, although the effect was clearly pleiotropic, as other signal transduction cascades (TGF-β, MAPK, and oxidative stress, the latter through its antioxidant properties) were in play [78]. A distinct mode of inhibition of Wnt signaling by dioscin was reported in an osteoarthritis mouse model, where mRNA expression of Wnt3a and β-catenin, sharply increased upon induction of the disease by iodoacetate, were counteracted by the compound treatment. In the study, dioscin was shown to also reduce oxidative ER stress not only through direct antioxidant activity but also by attenuation of Wnt pathway function, leading to a change in the activity of other pathways [11]. Finally, although somewhat contradictory, the effect of dioscin on the Wnt pathway in mouse osteoblast-like MC3T3-E1 and MG-63 cells was activating, with increased expression of LRP5 at mRNA and β-catenin at both mRNA and protein levels reported. This promoted osteoblastic proliferation and differentiation in both lines via OPG/RANKL induction. Curiously, this effect was inhibited by estrogen receptor antagonism, suggesting their cross-talk [6].

Finally, multiple and conflicting activities related to the Wnt pathway have been reported for the ginsenoside subfamily of steroid saponins. A ginsenoside derivative called 1C was shown to inhibit the Wnt pathway in LNCaP—a prostate cancer cell line—by decreasing the levels of β-catenin and TCF4, as well as target genes CCND1 and c-Myc, blocking its proliferation. The compound was also found to cause a sharp increase in ROS levels, which was found to be synergistic with its effects on Wnt, proving its role by counteracting with N-acylcysteine [79]. Ginsenoside Rg3 was also inhibitory by blocking the transcriptional activity of the pathway in colorectal cancer SW480 and HCT116 cell lines, the latter used in a xenograft model, where it showed therapeutic benefits. The authors used a TopFlash-Luc reporter system to assess activity and additionally examined the expression levels of c-Myc, a target gene, and showed that Rg3 treatment led to a significant decrease in nuclear β-catenin staining [80]. Beyond cancer, inhibition of the Wnt cascade by ginsenoside Rb1 was beneficial in adenine-induced chronic kidney disease in rats and in rat vascular smooth muscle cells as model systems involving vascular calcification, where it was also reported to be dependent on its antioxidant properties, providing a tantalizing possibility of cross-talk. The compound inhibited phosphorylation at Ser675 and nuclear translocation of β-catenin associated with calcification. In contrast, the Wnt pathway agonist SKL2001, which disrupts the β-catenin/axin interaction, potentiated this effect. It has been demonstrated that activation of the nuclear receptor PPAR-gamma is responsible for the anti-Wnt effect of the compound [3]. However, the exact same ginsenoside Rg1 exerted protective effects after intestinal ischemia/reperfusion injury by activating Wnt/β-catenin signaling. In vivo, pretreatment with the compound reduced apoptosis and inhibited ROS production, which was directly related to the activation of the Wnt pathway, as expressed by increased protein levels of Wnt1 and β-catenin, while both GSK3β and its phosphorylated form were reduced. DKK1, a specific antagonist of the pathway, blocked these effects on Wnt pathway components as well as the protective benefits of the compound, suggesting that these properties are mediated by activation of the pathway, rather than by its intrinsic antioxidant activity [19]. Another member of the ginsenoside family, F2, promoted hair growth by activating the pathway. In human hair dermal papilla cell line, it increased the expression of β-catenin and transcription factor LEF1, while levels of DKK1 decreased. In C57BL/6 mice, the compound caused an increase in the number of hair follicles and the thickness of the epidermis [22].

### 2.4. Ecdysteroids

**Figure 3:**
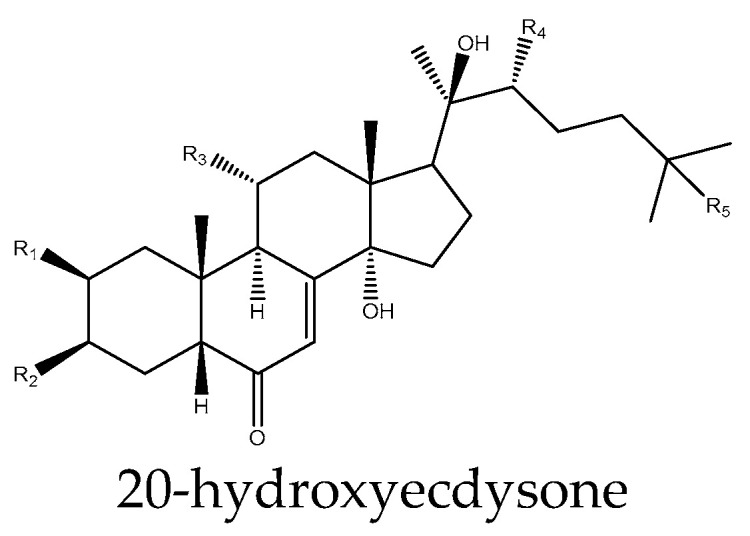
Example structure of an ecdysteroid 20-hydroxyecdysone reviewed in the current study.

Only two reports are available on the activity of this class of compounds in Wnt signaling, both dealing with pathway activation. β-Ecdysterone (20-hydroxyecdysone, Figure 3) was evaluated in the context of osteogenic differentiation in MC3T3-E1 preosteoblasts, where it caused significant enrichment of the gene signature associated with Wnt/β-catenin pathway activation and promoted differentiation in synergy with BMP-2 signaling [8]. This ecdysteroid has been reported to have potent neuroprotective effects due to its antioxidant properties, positively influenced the neuronal differentiation of adult rat hippocampal neural stem cells in vitro, and it consequently ameliorated learning and memory deficits induced by high-power microwave radiation in vivo by upregulating Wnt3a followed by increasing β-catenin [81].

### 2.5. Withanolides

Studies have shown that withanolides could be of potential use in anti-tumor agent development as a novel Wnt signaling inhibitors. Ref. [82] reported that 4β-hydroxywithanolide E, a natural withanolide from *Physalis peruviana*, increases the level of phosphorylated β-catenin and decreases the levels of active non-phosphorylated form and total β-catenin in colon cancer HCT116 cells. 4β-Hydroxywithanolide E has been shown to inhibit cell viability and induce cell cycle arrest at the G0/G1 phase and apoptosis in colon cancer cells, with minimal cytotoxicity against normal colonic epithelial cells (CCD-841-CoN). Moreover, in vivo, this compound dramatically inhibited tumor growth in HCT116 xenografts by attenuating the Wnt/β-catenin signaling pathway [82]. Withanolide B is an active component of *Withania somnifera* that can regulate the osteogenic differentiation of human bone marrow mesenchymal stem cells through the ERK1/2 and Wnt/β-catenin signaling pathways. When inhibitors of the ERK1/2 and Wnt/β-catenin signaling pathways were used, the enhancement of osteogenic differentiation induced by Withanolide B was attenuated. Withanolide B also effectively promoted bone healing in a rat tibial osteotomy model [83]. Steroid derivative physalin F isolated from *Physalis angulate* acts as an antagonist of Wnt/β-catenin signaling [84]. Their findings demonstrated for the first time that physalin F exhibited potential anti-tumor efficacy against colorectal cancer in vitro and in vivo by inhibiting Wnt/βcatenin signaling via accelerating the ubiquitination and degradation of β-catenin in a YAP-dependent manner.

### 2.6. Cucurbitacins

Cucurbitacins B and E have been shown to have Wnt inhibitory activity in four major cancers related to Wnt signaling. For example, cucurbitacin B was shown to be effective against lung cancer H1299 and A549 cell lines and against an in vivo mouse model induced by the tobacco carcinogen NNK, where it directly affected β-catenin and TCF1 interaction. The authors observed similar treatment effects in the cell lines when the pathway was inhibited by downregulating Wnt3 and Wnt3a [85]. In breast cancer T47D and SKBR-3 cell lines, the same compound prevented nuclear translocation of β-catenin and a mediator of this process, galectin-3, and it caused a loss of GSK3β phosphorylation. Overall, this resulted in the downregulation of Wnt target genes such as cyclin D1 and c-Myc and a decrease in TCF-driven luciferase TopFlash and subsequent inhibition of cancer cell growth [86]. Another cucurbitacin, E, inhibited proliferation of A549 (lung), Hep3B (hepatocellular), and SW480 (colon) cancer cell lines via downregulation of cell cycle regulators, including the Wnt target gene cyclin D1. This was achieved by preventing nuclear accumulation of β-catenin by inducing the tumor suppressor protein menin, a histone methyltransferase component previously shown to affect this process [87]. In another study, this compound improved the response of colorectal cancer cell lines DLD1 and HCT-116 to chemotherapy, as demonstrated by combined treatment with 5-FU. This was achieved primarily by downregulating two Wnt target genes, the xenobiotic efflux pumps ABCC1 and MDR1, together with reducing mRNA levels of other target genes such as PKM2, cyclin D1, Axin2, c-Myc and, additionally, β-catenin itself [88].

### 2.7. Triterpenes

Compounds of this class are very widely studied as modulators of the Wnt pathway, with a general dominance of inhibitory activity. Betulinic acid acted in this way in a pulmonary fibrosis model by reducing Wnt3a- or LiCl-induced transcriptional activity in a luciferase assay or at the level of several Wnt target genes in NIH-3T3 fibroblasts. In lung fibroblasts, either primary or immortalized Mlg lines, it suppressed nuclear accumulation of β-catenin and promoted its phosphorylation and degradation concomitant with loss of Wnt3a-induced LRP6 phosphorylation, with similar effects observed in vivo, exerting preventive effects in a bleomycin-induced mouse model of pulmonary fibrosis [23]. Two types of poricoic acid inhibit the Wnt pathway in proximal kidney epithelial HK-2 cells and MPC5 podocytes stimulated with TGF-β1 and Ang II, modeling chronic kidney disease. They suppressed the expression of Wnt1, β-catenin, and, particularly, its active, non-phosphorylated form. This led to a decline in the target genes Snail1, Twist, MMP-7, PAI-1, and Fsp-1, contributing to the attenuation of renal fibrosis [4]. Ganoderal A shows the opposite effect on Wnt signaling and promotes osteogenic differentiation of human amniotic mesenchymal stem cells by upregulating key pathway components such as β-catenin, Wnt3, and several FZD receptors, ultimately promoting osteogenic-specific proteins and genes. The study also shows that specific inhibition of the pathway by the Axin-targeting molecule KYA1797K at least partially blocked compound-induced differentiation, with the partiality likely due to the parallel involvement of BMP/SMAD signaling [9]. Triterpene corosolic acid has been identified as being an antagonist of the Wnt/β-catenin pathway in colon cancer cells [28].

Ursolic acid was found to be universally inhibitory in three studies. In the first, it mediated suppression of proliferation, migration, and clonality in CRC cell lines through reductions at the mRNA and protein levels in Wnt4, TCF4, LEF1, and β-catenin, including loss of nuclear translocation of the latter while increasing GSK3β [89]. In PC-3 prostate cancer cells, the compound showed cytotoxicity and increased apoptotic death. This was achieved via suppressing Wnt5a and b and β-catenin expression and increasing GSK3β phosphorylation at Ser9. The authors demonstrated the specificity of the activity, as GSK3β inhibitor SB216763 or Wnt3a prevented ursolic-acid-mediated caspase-3 and PARP cleavage [90]. Finally, in combination with corosolic acid, it attenuated the Wnt/β-catenin pathway in HEK293-FL reporter cells and HCT-15 colon cancer cells by promoting Wnt3a-induced β-catenin degradation through a b-TrCP-dependent proteasomal degradation pathway, resulting in inhibition of HCT-15 cell proliferation [28].

Oleanolic acid inhibited the Wnt pathway, as evidenced by a decrease in β-catenin in two reports. In primary rat chondrocytes induced by IL-1β, it additionally promoted GSK3β phosphorylation and decreased Wnt3a levels, as well as those of WISP1, a target gene, and crosstalk with Hippo/YAP, thus contributing to protective effects in osteoarthritis by preventing excessive ECM degradation [10]. In human hepatocellular carcinoma SMMC-7721 cells, in addition to low β-catenin both in the cytoplasm and nucleus by promoting its phosphorylation (Ser33/37/Thr41), the compound also caused target genes c-Myc and cyclin D1 to decrease, reducing a wide range of cancer properties [91].

**Figure 4:**
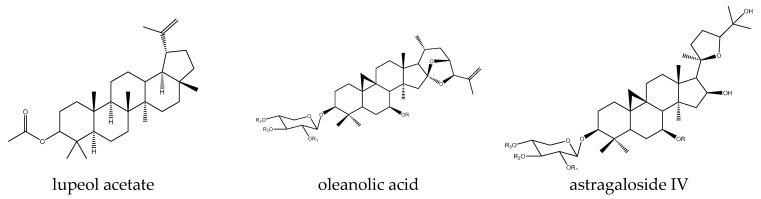
Example structures of triterpenes lupeol acetate, oleanolic acid, and astragaloside IV acetate reviewed in the current study.

Lupeol (Figure 4) inhibition of the Wnt/β-catenin pathway has been reported in at least four studies. In two of them, the compound caused a decrease in GSK3β phosphorylation: in the human hepatocellular carcinoma cell line Huh-7, it achieved this via PI3K/AKT, also suppressing LiCl-induced activity [92], while in prostate cancer LNCaP and DU145 cells, it also increased protein levels of Axin and reduced levels of target genes cyclin D1 and MMP-2 [93], in both cases leading to a loss of carcinogenicity. In colorectal cancer cells HCT 116 and DLD 1, it decreased cell viability, induced apoptosis, and reduced colonogenic potential but by different mechanisms: the compound prevented β-catenin translocation by affecting Ser552 and Ser675 phosphorylation and reduced transcriptional activity assessed by the Wnt target gene Axin2 and TCF1 expression. Its efficacy was lost when Wnt signaling was inhibited by dominant-negative TCF4 expression, as well as in a control RKO line with non-mutated β-catenin [94]. In melanoma cells with constitutive Wnt/β-catenin signaling, the compound decreased proliferation and viability both in vitro and in vivo. Efficacy was observed only in lines with APC or β-catenin mutations, which were responsive in a TopFlash reporter assay and by a reduction in target genes MITF, CRD-BP, and cyclin D1 and loss of β-catenin translocation. Similar effects were found in in vivo xenografts of these lines and in HEK293T reporter cells [95].

Finally, Wnt signaling effects have been reported for astragalosides in many, albeit conflicting, studies. For example, astragaloside I activates the Wnt/β-catenin and BMP signaling pathways in MC3T3-E1 cells, a model for osteoblast differentiation. This leads to upregulation of β-catenin and the target gene Runx2, which, in turn, causes an increase in the OPG/RANKL ratio, stimulating osteoblast differentiation and bone formation and potentially being useful in alleviating osteoporosis [96]. In contrast, the total astragaloside fraction negatively modulated Wnt signaling in the MO3.13 oligodendroglial cell line. There, it caused increased levels of p-GSK3β at Tyr216 and decreased at Ser9, both leading to increased phosphorylation of β-catenin as well as decreased expression of TCF4 and the target gene Id2, ultimately leading to increased transcriptional expression of neuronal myelin-binding protein (MBP). These observations were recapitulated in the corpus callosum of a CPZ-induced mouse model of demyelination, where the compound alleviated behavioral impairment, also related to its antioxidant activity [18].

Contradictory effects have been reported for even a single member of this family. For example, in four reports, astragaloside IV was found to activate the Wnt/β-catenin signaling pathway, which, in all cases, was generally detected by increased β-catenin stability. In bovine mammary epithelial cells, activation of the pathway by the compound antagonized its LPS-induced inflammatory-associated inhibition mediated by ROS accumulation. Moreover, astragaloside IV did not exert any direct radical scavenging activity but only counteracted ROS-induced effects through pathway activation, as also evidenced by an increase in target gene expression, with β-catenin silencing results confirming its specific role as a pathway activator [97]. The compound promoted osteogenesis of bone-marrow-derived mesenchymal stem cells through pathway activation, achieving its effect on β-catenin via a reduction in GSK3β Ser9 phosphorylation and in cross-talk with nerve growth factor NGF [98]. It was found to enhance osteogenic differentiation in rat tibia defect models and rat bone marrow mesenchymal stem cells through NMUR2 crosstalk, with the response to the compound reversed in the presence of the inhibitor DKK1 [99]. Finally, it can be used to protect against intestinal dysfunction induced by peritoneal dialysis both in vitro in T84 cells and in vivo in peritoneal dialysis mouse models by activating the pathway through increasing GSK3β phosphorylation levels [20].

In contrast, in six other studies, astragaloside IV was found to exert inhibitory activity on the Wnt pathway, with the only common feature among them being a decrease in β-catenin levels between them, with different levels of detail addressed experimentally in these studies. In this way, the compound suppressed the epithelial–mesenchymal transition, which was also partly achieved through direct ROS scavenging activity and associated carcinogenic properties of hepatocellular carcinoma Huh7 and MHCC97-H cells, which was caused by controlling the phosphorylating activity of AKT via Ser9 in GSK3β, as confirmed by the finding that the AKT inhibitor GSK690693 was effective in suppressing it [100]. In hepatocellular carcinoma SMMC7721 and Huh7 lines, this was achieved by miR-150-5p action and caused their apoptosis, with β-catenin overexpression counteracting this effect. In vivo assays confirmed the drug-induced reduction in tumor size, an effect that was also neutralized by β-catenin overexpression [101]. The compound prevented the TGF-β1-induced activation of the Wnt pathway during the epithelial–mesenchymal transition in U251 glioma cells, reversing the expression of N-cadherin, vimentin, and cyclin-D1, with overexpression of β-catenin causing these effects of the compound to be counteracted [102]. It also antagonized fibrosis in a rat model of unilateral ureteral obstruction through a Wnt-inhibitory mechanism in addition to its antioxidant-related action. In addition to the above-mentioned β-catenin decrease, the compound caused a drop in the levels of Wnt3 and Wnt4, FZD4, and p-LRPs, and it reduced the expression of transcription factors LEF1 and TCF1 and the target genes Snail, Jagged, E-cadherin, Twist, MMP2, and MMP7 [24]. Finally, LiCl is known to demonstrate impaired re-epithelialization in mouse keratinocytes by reducing their proliferation and migration through unwanted Wnt activation, with astragaloside IV able to suppress β-catenin expression in these conditions [25].

### 2.8. Iridoids

A strong indication of the ability of many of these compounds to affect Wnt signaling comes from testing a collection of iridoid-inspired derivatives obtained via an asymmetric catalytic method. They were evaluated for their effect on the Wnt and Hedgehog signaling pathways using HEK293 reporter cell lines. From a total of 115 derivatives, 20 specific inhibitors of the Wnt pathway were identified, and 6 additional compounds inhibited the Hedgehog pathway [103]. It is thus not surprising to see that several naturally sourced compounds of this family have been reported to have similar activity, and it should be noted that a large proportion of these compounds have been reported as Wnt enhancers. Thus, using the TNFalpha-stimulated MH7A cell line and a rat model of collagen-induced arthritis, the mechanism of the beneficial effect of penta-acetyl geniposide was confirmed by the authors in that, both in vivo and in vitro, the compound reduced the levels of Wnt1, p-GSK3β, and β-catenin, and it inhibited the nuclear translocation of the latter. The authors also used XAV939, a tankyrase-targeting Wnt inhibitor, to mimic the effects of the compound, confirming the specific role of Wnt inhibition in this process [12]. On the other hand, iridoid cornin was beneficial in a rat model of stroke by enhancing angiogenesis and improving functional recovery. In vitro, on an arterial smooth muscle cell line, it increased their proliferation and, thus, angiogenesis by enhancing the expression of Wnt5a, β-catenin, and target gene cyclin D1, and it is also known to exert similar effects partly due to its antioxidant properties [14].

Conflicting activities have been reported even for the same compound. Catalpol had an inhibitory activity on the Wnt/β-catenin pathway in the context of renal fibrosis. In a model of this condition in rats, where the pathway was aberrantly activated, it counteracted this process by reducing levels of Wnt3a, p-GSK3β, and β-catenin and improved outcomes. It also reversed TGF1-induced upregulation of Snail1, a Wnt target gene, and attenuated EMT in the proximal tubular line HK-2 [5]. Conversely, in bone marrow mesenchymal stem cells, catalpol treatment enhanced the expression of β-catenin and the Wnt transcription factors LEF1 and TCF1/7 and increased the levels of phosphorylated GSK3β and nuclear β-catenin. Selectivity was controlled by experiments with DKK1, a natural Wnt inhibitor, which reversed these effects, albeit partly apparently due to additional effects from the MAPK and BMP pathways, which were also upregulated [104].

**Figure 5:**
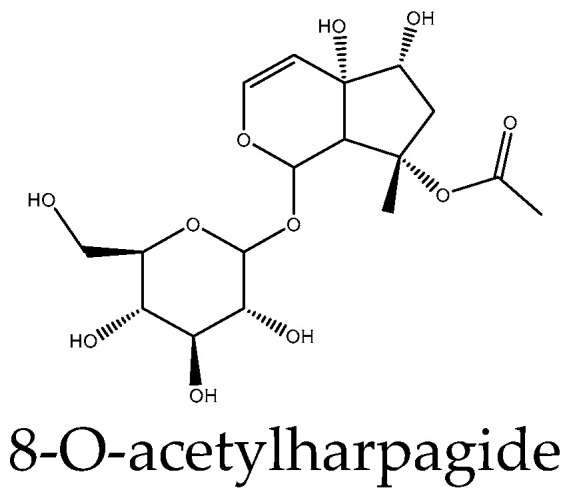
Example structure of iridoid 8-O-acetylharpagide reviewed in the current study.

The rest of the available reports deal only with the activating activity of the compounds on the Wnt pathway. Harpagoside induced the differentiation of the osteoblast line MC3T3-E1 by increasing the protein expression of a secreted Wnt inhibitor DKK1, the Wnt target genes cyclin D1 and c-Myc, and β-catenin and suppressing the phosphorylation of the latter [105]. Its close derivative harpagide (Figure 5) attenuated neuronal apoptosis after spinal cord injury in a rat model by increasing the expression levels of β-catenin, which was controlled by observing the loss of the protective effect of harpagide upon inhibition of Wnt by the tankyrase inhibitor XAV939 [17]. Finally, two reports concern morronside. In the first, it might be expected to enhance hair growth via an effect on outer root sheath cells in vitro by upregulation of Wnt10b, β-catenin, and LEF1—effects counteracted by DKK1 [106]. Morroniside has neurorestorative effects and promotes neurogenesis, providing improvements in the context of ischemic stroke. In a rat model of middle cerebral artery occlusion, the compound caused increased expression of Wnt3a, β-catenin, and TCF4, as well as neurogenesis-related target genes of Wnt, the transcription factors Ngn2 and Pax6 [15].

Harpagoside treatment induces osteoblast differentiation via upregulating the expression of β-catenin and cyclin D1 as well as c-Myc and downregulating Dkk1 expression [105]. Takayama et al. [107] synthesized collections of iridoid-inspired compounds and investigated their bioactivity using HEK293 reporter cell line stimulated by Wnt3a. The SAR analysis suggested that groups connected via C-1 are important for Wnt pathway inhibition activity. The bulky carboxylic residues connected to C-1 decreased their Wnt inhibitory activities, whereas compounds with acetic acid ester in the C-1 position generally showed potent Wnt inhibition activity.

### 2.9. Terpenoids

The natural diterpene jatrophone isolated from *Jatropha isabelli* and *Jatropha gossypiifolia* interferes with Wnt/β-catenin signaling and inhibits proliferation and EMT in human triple-negative breast cancer [33,34]. Andrographolide, a labdane diterpenoid produced by the plant *Andrographis paniculata*, is a potent Wnt signaling activator that acts by inhibiting GSK3β by a non-ATP-competitive, substrate-competitive mode of action [108]. Euphorbiaceae plants are otherwise famous for phorbol and its esters, and the results showed that they produced strong Wnt-inhibiting activities in triple-negative breast and colon cancer cells. Also, natural and semisynthetic tigliane diterpenoids from the plant *Euphorbia dracunculoides* inhibited Wnt signaling in a luciferase assay in HEK293 cells, reducing the expression of Wnt target genes Axin2, c-myc, and cyclin D; phosphorylation and degradation of β-catenin were similarly observed in HEK293W cells incubated with tigliane diterpenoids [35,109], identifying shizukaol D, a dimeric sesquiterpene, as a β-catenin signaling inhibitor. It has been found that shizukaol D inhibits cell viability and colony formation and induces apoptosis in liver cancer cells by downregulating the expression of β-catenin and its upstream regulators LRP, Dvl2, and Axin2. The activation of β-catenin by wnt3a blocks shizukaol-D-induced cell growth inhibition, indicating that the β-catenin signaling plays a critical role in the anti-cancer activity of this compound.

### 2.10. Coumarins

**Figure 6:**
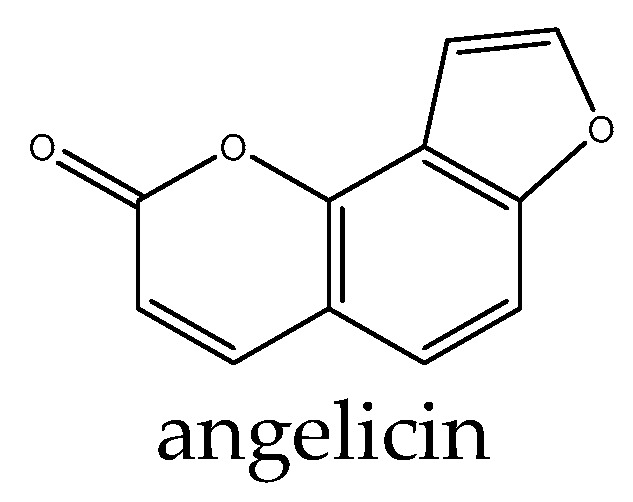
Example structure of coumarin angelicin reviewed in the current study.

Studies have investigated the potential of coumarin derivatives as therapeutic agents targeting the Wnt signaling pathway. Pelusi et al. [110] found that the coumarin derivative umbelliferon significantly increased the expression of β-catenin, and this effect was significantly reverted by 5 µM tamoxifen. In addition, umbelliferon-treated cells showed a significant increase in β-catenin protein levels, which were significantly reduced following 24 and 72 h tamoxifen treatment, suggesting that the role exerted by umbelliferon in cultured hOBs can be mediated by the ESR1/β-catenin pathway. Angelicin is a natural coumarin compound in *Psoralea corylifolia* that promoted the expression of β-catenin and runt-related transcription factor 2, which serve a vital role in the Wnt/β-catenin signaling pathway. Consistently, the osteogenic effect of angelicin was attenuated by the use of a Wnt inhibitor. Moreover, angelicin (Figure 6) increased the expression of estrogen receptor α (ERα), which also serves a key role in osteoblast differentiation [111].

### 2.11. Flavonoids and Phenolics

The regulation of the Wnt pathway by various flavonoids, in particular quercetin apigenin, daidzein, quercitrin, naringenin, rutin, myricetin, epigallocatechin, catechin, isorhamnetin, kempferol, and baicalein, has been evaluated in many studies and has been excellently reviewed elsewhere [123], therefore we refer the reader to this review and do not see the need to unnecessarily repeat its conclusions in the present work.

**Figure 7:**
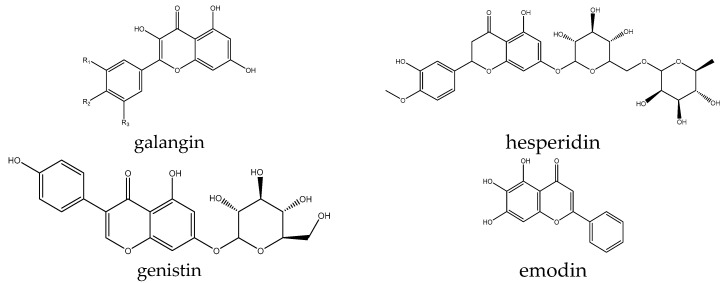
Example structures of flavonoids galangin, emodin, genistin, and hesperidin reviewed in this study.

However, some additional examples of flavonoid-type compounds have been shown to have effects on Wnt signaling, both activating and inhibiting. Several compounds were reported in only a single paper, with two of them reported as signaling activators. For example, the effect of galangin (Figure 7) was evaluated in a stroke model based on middle cerebral artery occlusion in rats. The authors found a decrease in the mRNA levels of β-catenin, LRP6, and FZD1 as well as a typical pathway inhibition signature of a reduction in p-GSK3β and an increase in phospho-β-catenin, thus reversing the pathogenic activation observed in the model [16]. Hesperidin promoted osteogenic differentiation of human alveolar osteoblasts partly by the scavenging of ROS induced by high glucose as well as through Wnt signaling, as evidenced by increased levels of target genes such as RUNX2 and cyclin D1, and it also increased levels of β-catenin. The effects of the compound were confirmed to be specific and reversed by the inhibitor DKK1 [112]. In contrast, genistin inhibited the pathway in glioma U-87 cells, decreased the levels of Wnt1 and Wnt3a, and increased the p-GSK3β/GSK3β ratio. This resulted in the suppression of cell viability and proliferation in this line [113].

Formononetin or its close derivatives were evaluated in several experimental setups and showed somewhat contradictory results. Thus, it increased the transcriptional levels of Wnt10b and LRP5, promoted nuclear accumulation of β-catenin and phosphorylated levels of Ser9 of GSK3β, and decreased DKK2 expression and in 3T3-L1 pre-adipocytes. This pathway activation was associated with a surprising increase in ROS by this normally antioxidant compound, leading to a specific pathway activation by AMPK, which was beneficial against bone loss and obesity [13]. Its sulfonated derivative significantly increased the expression levels of Wnt5a, β-catenin, and cyclin D1 in human umbilical vein endothelial cells, contributing to angiogenesis. The activating effects were specifically attenuated by the pathway antagonists WIF1 and DKK1 [114]. However, in contrast to these two reports, formononetin decorated with a dithiocarbamate group inhibited the migration of PC-3 cells and negatively regulated the Wnt signaling pathway by increasing the expression of Axin and decreasing β-catenin and TCF4 [115].

Finally, a large body of data was accumulated on the Wnt inhibitory activity of emodin. The compound counteracted ischemia-induced Wnt activation through a reduction in β-catenin in a rat retinal ischemia model, also involving HIF-1α and VEGF, and it had an overall protective effect in the model [26]. A reduction in GSK3β Ser9 phosphorylation and decrease in β-catenin levels as well as EMT-related Wnt target genes in response to the compound were found in a separate study in HepG2 hepatocellular carcinoma [124] and in A2780 and SK-OV-3 epithelial ovarian cancer lines, inhibiting EMT in these lines [116]. Highly similar to emodin, its isomer by a single OH group, aloe-emodin has similar activities, and it reduced the proliferation, migration, and invasion of melanoma cell lines A375 and SK-MEL-28 in an in vivo model based on them by modulating the Wnt/β-catenin signaling pathway by reducing Wnt3a, increasing the p-β-catenin/β-catenin ratio, and reducing p-GSK3β. Overexpression of β-catenin in in vivo xenografts reversed the effects of the compound on tumor growth, confirming the specificity [117]. Aloe-emodin decreased the expression of β-catenin and the target genes cyclin D1 and c-Myc, causing pro-oxidant effects and elevated ROS, and it thus inhibited the growth of androgen-independent DU145 prostate cancer cells. Molecular docking analysis suggested direct binding of the compound to β-catenin and Wnt2 [118]. Finally, a derivative 4-aminoethylamino-emodin was identified as a direct inhibitor of GSK3β, however with minor effects on the Wnt transcriptional readout, since the reduction in β-catenin phosphorylation levels by this compound was competed by a simultaneous second activity of the compound locking this protein outside the free cytoplasmic pool [119].

### 2.12. N-Containing Compounds

The most frequently accumulating nitrogen-containing compounds in plants include amino acids, amides, amino acids, proteins, quaternary ammonium compounds, and polyamines.

Nature is rich in nitrogen compounds, with numerous examples found in plants, often manifesting as alkaloids. A pyridone alkaloid ricinine isolated from *Ricinus communis* showed the ability to stimulate the Wnt cascade, having CK1 as the target [36]. Berberine, a benzylisoquinoline plant alkaloid from *Coptidis rhizoma*, promoted osteogenic differentiation of bone mesenchymal stem cells, concomitant with accumulation of total and nuclear β-catenin; activation of the Wnt cascade was also confirmed by a TopFlash assay. Furthermore, the Wnt signaling inhibitor DKK1 was blocked by berberine [38]. Also, the plant-derived isoquinoline alkaloid berberine and its synthetic 13-arylalkyl derivatives reduced the levels of cytoplasmic β-catenin in HCT116 human colon carcinoma cells [120]. Matrine, a class of tetracyclic quinoline alkaloids extracted from plants in the genus *Sophora*, inhibited the expression of VEGF and the proliferation of breast cancer cells by regulating the Wnt/β-catenin signaling pathway [37]. Some matrine derivatives can also inhibit the proliferation of hepatocellular carcinoma (HCC) cells through the PI3K/AKT/mTOR and AKT/GSK3/β-catenin signaling pathway [121].

## 3. Conclusions and Perspectives

Natural compounds have had an immense impact as a source of novel therapeutics, with up to half of recently approved drugs inspired by nature. This is largely due to the diversity of their structures, with modifications that are often difficult to achieve through synthetic methods [125]. These compounds have the potential to modulate multiple targets and pathways, and they often possess antioxidant properties that are intricately intertwined with cellular signaling cascades. This gives them the ability to address complex diseases by restoring cellular homeostasis and mitigating disease progression, also demonstrated by the ensemble of studies reviewed here, which encompass a wide variety of different models and conditions [125,126].

However, significant improvements are needed to close the gap in translating these findings into actual treatments. Often, studies rely on indirect measures of pathway modulation rather than specific molecular interactions, focusing on elucidating downstream effects rather than underlying mechanisms. In particular, reliance on a narrow range of assays, such as GSK3β phosphorylation, β-catenin stabilization, and qPCR-based assessment of multiple target gene expression, often provides an incomplete picture of the complex and nuanced interplay between antioxidants and pathways. A panel of tailored orthogonal readouts that independently assess different aspects of the Wnt pathway and crosstalk with RO/NS and that preferably explore molecular mechanisms of action should be employed to improve the validity of studies on natural compounds [127]. Few of the studies reviewed here provide such detailed results [21,28,62,84,108]. In silico modeling and bioinformatics analyses, such as network pharmacology approaches, deserve to be better exploited for predicting potential targets and mechanisms to guide experimental design [128,129,130].

Discrepancies in compound effects, up to completely opposite effects reported for the same compound, can be addressed by rigorous evaluation of the ADME properties of compounds used in animal models, as differences in pharmacokinetics or active metabolites may be behind such differences [131]. This is of particular importance, as many promising natural antioxidant compounds suffer from poor solubility, stability, and bioavailability, hindering their clinical translation. This can be overcome not only by chemical derivation [132,133] but also by innovative delivery systems such as liposomes, polymeric nanoparticles, and nanocarriers [134], which have the potential to enhance drug solubility [135], protect compounds from degradation, prolong circulation time, and improve cellular uptake [131].

Indicated venues will be instrumental in unlocking the full therapeutic potential of natural antioxidant compounds for the benefit of human health. Future research should strive for a holistic approach for the comprehensive development of these promising therapeutic agents.

## Figures and Tables

**Table 1:** Summary table describing the direction, mechanism of action, and experimental system used for each compound in this review.

Compound		Wnt Signaling Influence	Experimental System	Ref.
**2.2. Cardiac glycosides**	Ouabain	Inhibition (reduces β-catenin nuclear translocation)	Esophageal cancer cell line EC109	[53]
		Activation (promotes β-catenin nuclear shuttle via tyrosine phosphorylation)	MDCK cells	[54]
		Activation (inactivates GSK3β, leading to β-catenin stabilization and nuclear translocation)	Hippocampus of adult rats	[55]
	Oleandrin	Inhibition (suppresses TopFlash reporter and target genes, inhibits β-catenin nuclear translocation)	Osteosarcoma cell lines U2OS and SaOS-2	[56]
		Inhibition (suppresses TopFlash reporter and target genes, inhibits β-catenin nuclear translocation)	Breast cancer cell line MDA-MB-231 and its radioresistant subclone	[57]
	Odoroside A	Inhibition (contributes to reduced invasiveness and radioresistance, partly through decreased β-catenin levels)	Breast cancer cell line MDA-MB-231 and its radioresistant subclone	[57]
	Peruvoside	Inhibition (decreases β-catenin levels in nucleus and cytoplasm, reduces expression of Wnt target genes)	MCF-7 breast cancer, A549 lung cancer, and HepG2 liver cancer cell lines	[58]
	Strophant-hidine	Inhibition (decreases β-catenin levels in nucleus and cytoplasm, reduces expression of Wnt target genes)	MCF-7 breast cancer, A549 lung cancer, and HepG2 liver cancer cell lines	[59]
	Nerigoside	Inhibition (suppresses β-catenin degradation via ERK crosstalk, promoting GSK3β degradation)	Colorectal cancer cell lines HT29 and SW620	[60]
	H-9	Inhibition (downregulates Wnt signaling through direct interaction and induction of β-catenin degradation)	Colorectal cancer cells	[61]
	ATE-i2-b4			[61]
	Convalla-toxin	Inhibition (through crosstalk with parathyroid hormone receptor 1 (PTHR1))	Osteosarcoma cells	[62]
	Calotropin	Inhibition (increases CK1a levels leading to increased β-catenin degradation)	Colon cancer cells	[63]
	Bufalin	Activation (inhibits AKT phosphorylation, potentially leading to decreased GSK3β activity and increased β-catenin stability)	Hepatoma cell lines	[64]
		Inhibition (suppresses CCRK-driven transcription, leading to loss of active β-catenin)	Liver cancer	[65]
	Telocino- bufagin	Inhibition (inhibits Wnt/β-catenin signaling via modulation of GSK3β activity and by negatively affecting β-catenin stability, with lower potency for marinobufagin)	LLC-PK1 porcine kidney epithelial cells	[2]
	Marino-bufagin			
**2.3. Steroid saponins**	Ophiopo-gonin B	Inhibition (reduces β-catenin, cyclin D1, c-Myc, strengthens Axin/β-catenin interaction)	Non-small-cell lung carcinoma cell lines	[66]
	Epi-brassinolide	Inhibition (reduces β-catenin, c-Myc, CCND1, Sox9, c-Jun, survivin, MMP1, MMP7, mPar)	NCI-H69 and VPA17 small-cell lung carcinoma line	[67]
	Saponin (Tupistra)	Inhibition (reduces β-catenin and c-Myc)	Ovarian cancer cells SKOV3	[68]
	Daucosterol	Inhibition (reduces β-catenin and Wnt5a, increases GSK3β)	Hepatocellular carcinoma lines HepG2 and SMMC-7721	[69]
	Paris saponin H	Inhibition (downregulates β-catenin and p-GSK3β)	Hepatocellular carcinoma PLC/PRF/5 and Huh7 cells, in vivo xenograft	[70]
	Paris saponin I	Inhibition (decreases β-catenin)	Zebrafish	[71]
	Polyphyllin I (Chong Lou)	Inhibition (suppresses p-GSK3β, decreases β-catenin)	Osteosarcoma cell lines (in vitro), xenograft orthotopic mouse model	[72]
		Inhibition (suppresses p-GSK3β, decreases β-catenin, requires AKT crosstalk)	Liver cancer stem cells	[73]
		Inhibition (counteracts aberrant Wnt activation by AngII, decreases β-catenin, p-GSK3β, c-Myc, c-Jun, c-Fos, cyclin D1)	Mouse model of pressure overload-induced cardiac hypertrophy, cultured neonatal rat ventricular myocytes	[21]
	Diosgenin	Inhibition (decreases β-catenin)	MG-63 osteoblast-like cells	[74]
		Inhibition (decreases β-catenin)	HCT-116 human colon carcinoma cells	[75]
		Inhibition (upregulates sFRP4 and DKK1)	Breast cancer cell lines (mammosphere-enriched)	[76]
	Timosapo-nin AIII	Inhibition (decreases β-catenin, cyclin D1, c-Myc, crosstalk with AKT)	Human leukemia HL-60 cells	[77]
	Timosaponin B II	Activation (increases Dishevelled, increases β-catenin)	UMR-106 osteoblastic cells and primary rat osteoblasts	[7]
	Dioscin	Inhibition (increases GSK3β activity by decreased phosphorylation, downregulates nuclear β-catenin, blocks AKT)	Osteosarcoma model, xenograft tumor	[69]
		Inhibition (increases GSK3β activity by decreased phosphorylation, downregulates nuclear β-catenin)	Rat model of CCl4-induced liver fibrosis, primary rat hepatic stellate cells (in vitro and in vivo)	[78]
		Inhibition (counteracts increased Wnt3a and β-catenin)	Osteoarthritis mouse model	[11]
		Activation (increases LRP5 mRNA, β-catenin mRNA and protein)	Mouse osteoblast-like MC3T3-E1 and MG-63 cells	[6]
	Ginsenoside 1C	Inhibition (decreases β-catenin, TCF4, CCND1, c-Myc)	LNCaP prostate cancer cell line	[79]
		Inhibition (blocks transcriptional activity, decreases nuclear β-catenin, c-Myc)	Colorectal cancer cells SW480 and HCT116 cell lines, xenograft model	[80]
		Inhibition (inhibits p-β-catenin Ser675 and nuclear translocation)	Adenine-induced chronic kidney disease in rats, rat vascular smooth muscle cells	[3]
		Activation (increases Wnt1 and β-catenin, reduces GSK3β and p-GSK3β)	Mouse model of intestinal ischemia–reperfusion injury (in vivo), DKK1 blocking experiments	[19]
		Activation (increases β-catenin and LEF1, decreases DKK1)	Human hair dermal papilla cell line, C57BL/6 mice	[22]
**2.4. Ecdysteroids**	β-Ecdysterone (20-hydroxyecdysone)	Activation (enriched gene signature of Wnt/β-catenin activation, synergy with BMP-2)	MC3T3-E1 preosteoblasts	[8]
		Activation (upregulates Wnt3a followed by increasing β-catenin)	Adult rat hippocampal neural stem cells (in vitro), rat model of microwave-radiation-induced learning and memory deficits (in vivo)	[81]
**2.5. Withanolides**	4β-hydroxy-withanolide E	Inhibition (increases phosphorylated β-catenin, decreases active and total β-catenin)	Colon cancer HCT116 cells, HCT116 xenografts (in vivo)	[82]
	Withanolide B	Potentiation/activation (enhances osteogenic differentiation through Wnt/β-catenin pathway)	Human bone marrow mesenchymal stem cells (in vitro), rat tibial osteotomy model (in vivo)	[83]
	Physalin F	Inhibition (antagonist of Wnt/β-catenin signaling, accelerates β-catenin ubiquitination and degradation in a YAP-dependent manner)	Colorectal cancer cells (in vitro and in vivo)	[84]
**2.6. Cucurbitacins**	Cucurbitacin B	Inhibition (directly affects β-catenin and TCF1 interaction)	Lung cancer H1299 and A549 cell lines, in vivo mouse model (NNK-induced)	[85]
		Inhibition (prevents β-catenin nuclear translocation, loss of p-GSK3β, downregulates cyclin D1 and c-Myc)	Breast cancer T47D and SKBR-3 cell lines	[86]
	Cucurbitacin E	Inhibition (prevents β-catenin nuclear accumulation by inducing menin, downregulates cyclin D1)	Lung (A549), hepatocellular (Hep3B), and colon (SW480) cancer cell lines	[87]
		Inhibition (downregulates ABCC1, MDR1, PKM2, cyclin D1, Axin2, c-Myc, and β-catenin combined with 5-FU)	Colorectal cancer cell lines DLD1 and HCT-116	[88]
**2.7. Triterpenes**	Betulinic acid	Inhibition (reduces Wnt3a-/LiCl-induced transcription, suppresses β-catenin nuclear accumulation, promotes β-catenin phosphorylation and degradation, loss of p-LRP6)	NIH-3T3 fibroblasts, primary and immortalized lung fibroblasts (Mlg line), bleomycin-induced mouse model of pulmonary fibrosis (in vivo)	[23]
	Poricoic acid	Inhibition (suppresses Wnt1, β-catenin, active β-catenin, Snail1, Twist, MMP-7, PAI-1, Fsp-1)	Proximal kidney epithelial cells HK-2 and podocytes MPC5 (stimulated with TGF-β1 and Ang II)	[4]
	Ganoderal A	Activation (upregulates β-catenin, Wnt3, FZD receptors, osteogenic-specific proteins and genes)	Human amniotic mesenchymal stem cells	[9]
	Corosolic acid	Inhibition (antagonist of Wnt/β-catenin pathway)	Colon cancer cells	[28]
	Ursolic acid	Inhibition (reduces Wnt4, TCF4, LEF1, β-catenin, inhibits β-catenin nuclear translocation, increases GSK3β)	CRC cell lines	[89]
		Inhibition (suppresses Wnt5a/b and β-catenin, increases p-GSK3β Ser9)	PC-3 prostate cancer cells	[90]
		Inhibition (promotes Wnt3a-induced β-catenin degradation via b-TrCP)	HEK293-FL reporter cells, HCT-15 colon cancer cells	[28]
	Oleanolic acid	Inhibition (decreases β-catenin, promotes p-GSK3β, decreases Wnt3a, WISP1)	Primary rat chondrocytes (induced by IL-1β)	[10]
		Inhibition (low β-catenin in cytoplasm and nucleus by promoting phosphorylation, decreases c-Myc and Cyclin D1)	Human hepatocellular carcinoma SMMC-7721 cells	[91]
	Lupeol	Inhibition (decreases p-GSK3β via PI3K/AKT, suppresses LiCl-induced activity)	Human hepatocellular carcinoma cell line Huh-7	[92]
		Inhibition (decreases p-GSK3β, increases Axin, reduces cyclin D1 and MMP-2)	Prostate cancer LNCaP and DU145 cells	[93]
		Inhibition (prevents β-catenin translocation by affecting Ser552 and Ser675 phosphorylation, reduces Axin2 and TCF1 expression)	Colorectal cancer cells HCT 116 and DLD 1	[94]
		Inhibition (decreases proliferation and viability, reduces MITF, CRD-BP, cyclin D1, loss of β-catenin translocation)	Melanoma cells with APC or β-catenin mutations, in vivo xenografts, HEK293T reporter cells	[95]
	Astra-galoside I	Activation (upregulates β-catenin and Runx2, increases OPG/RANKL ratio)	MC3T3-E1 cells	[96]
	Total astragaloside fraction	Inhibition (increases p-GSK3β Tyr216, decreases p-GSK3β Ser9, decreases TCF4 and Id2)	MO3.13 oligodendroglial cell line, CPZ-induced mouse model of demyelination (corpus callosum)	[18]
	Astragaloside IV	Activation (increases β-catenin stability, antagonizes LPS-induced inhibition)	Bovine mammary epithelial cells	[97]
		Activation (promotes osteogenesis, reduces p-GSK3β Ser9, crosstalk with NGF)	Bone-marrow-derived mesenchymal stem cells	[98]
		Activation (enhances osteogenic differentiation, NMUR2 crosstalk, reversed by DKK1)	Rat tibia defect models and rat bone marrow mesenchymal stem cells	[99]
		Activation (increases p-GSK3β levels)	T84 cells (in vitro), peritoneal dialysis mouse models (in vivo)	[20]
		Inhibition (suppresses EMT, controls AKT phosphorylation via p-GSK3β Ser9)	Hepatocellular carcinoma Huh7 and MHCC97-H cells	[100]
		Inhibition (via miR-150-5p action, β-catenin overexpression counteracts)	Hepatocellular carcinoma SMMC7721 and Huh7 lines, in vivo assays	[101]
		Inhibition (prevents TGF-β1-induced Wnt activation, reverses N-cadherin, vimentin, cyclin-D1, β-catenin overexpression counteracts)	U251 glioma cells	[102]
		Inhibition (antagonizes fibrosis, decreases β-catenin, Wnt3, Wnt4, FZD4, p-LRPs, LEF1, TCF1, Snail, Jagged, E-cadherin, Twist, MMP2, MMP7)	Rat model of unilateral ureteral obstruction	[24]
		Inhibition (suppresses β-catenin expression)	Mouse keratinocytes (LiCl-induced impaired re-epithelialization model)	[25]
**2.8. Iridoids**	Iridoid-inspired	Inhibition (20 derivatives)	HEK293 reporter cell lines	[103]
	Penta-acetyl geniposide	Inhibition (reduces Wnt1, p-GSK3β, β-catenin, inhibits β-catenin nuclear translocation)	TNFalpha-stimulated MH7A cell line (in vitro), rat model of collagen-induced arthritis (in vivo)	[12]
	Cornin	Activation (enhances Wnt5a, β-catenin, cyclin D1)	Arterial smooth muscle cell line (in vitro), rat model of stroke (in vivo)	[14]
	Catalpol	Inhibition (reduces Wnt3a, p-GSK3β, β-catenin, Snail1)	Rat model of renal fibrosis, proximal tubular line HK-2	[5]
	Catalpol	Activation (enhances β-catenin, LEF1, TCF1/7, increases p-GSK3β, nuclear β-catenin)	Bone marrow mesenchymal stem cells	[104]
	Harpa-goside	Activation (increases DKK1, cyclin D1, c-Myc, β-catenin, suppresses p-β-catenin)	Osteoblast line MC3T3-E1	[105]
	Harpagide	Activation (increases β-catenin)	Rat model of spinal cord injury (in vivo)	[17]
	Morroniside	Activation (upregulates Wnt10b, β-catenin, LEF1)	Outer root sheath cells (in vitro)	[106]
		Activation (increases Wnt3a, β-catenin, TCF4, Ngn2, Pax6)	Rat model of middle cerebral artery occlusion (in vivo)	[15]
	Harpa-goside	Activation (upregulates β-catenin, cyclin D1, c-Myc, downregulates Dkk1)	Osteoblast line MC3T3-E1	[105]
	Iridoid-inspired	Inhibition (potency related to C-1 substituents)	HEK293 reporter cell line stimulated by Wnt3a	[107]
**2.9. Terpenoids**	Jatrophone	Inhibition (interferes with Wnt/β-catenin signaling)	Human triple-negative breast cancer cells	[34]
	Andrographolide	Activation (inhibits GSK3β)	Primary hippocampal neuron cultures, in vivo in rats	[108]
	Tigliane diterpenoids	Inhibition (reduces Axin2, c-myc, cyclin D, promotes β-catenin phosphorylation and degradation)	HEK293 and HEK293W cells (luciferase assay)	[35,109]
	Shizukaol D	Inhibition (downregulates β-catenin, LRP, Dvl2, Axin2)	Liver cancer cells	[109]
**2.10. Coumarins**	Umbelli-feron	Activation (increases β-catenin, ESR1/β-catenin pathway)	Cultured hOBs	[110]
	Angelicin	Activation (promotes β-catenin and runt-related transcription factor 2, increases ERα)	Human osteoblast culture	[111]
**2.11. Flavonoids and phenolics**	Galangin	Inhibition (decreases β-catenin, LRP6, FZD1, reduces p-GSK3β, increases p-β-catenin)	Rat model of middle cerebral artery occlusion (stroke model)	[16]
	Hesperidin	Activation (increases RUNX2, cyclin D1, β-catenin)	Human alveolar osteoblasts	[112]
	Genistin	Inhibition (decreases Wnt1 and Wnt3a, increases p-GSK3β/GSK3β ratio)	Glioma U-87 cells	[113]
	Formo-nonetin	Activation (increases Wnt10b and LRP5, promotes nuclear β-catenin, p-GSK3β Ser9, decreases DKK2 via AMPK)	3T3-L1 pre-adipocytes	[13]
	Sulfonated formononetin	Activation (increases Wnt5a, β-catenin, cyclin D1)	Human umbilical vein endothelial cells	[114]
	Formononetin + dithiocarbamate	Inhibition (increases Axin, decreases β-catenin and TCF4)	PC-3 cells	[115]
	Emodin	Inhibition (reduces β-catenin)	Rat retinal ischemia model	[26]
		Inhibition (reduces p-GSK3β Ser9, β-catenin, EMT-related Wnt target genes)	HepG2 hepatocellular carcinoma	[69]
		Inhibition (reduces p-GSK3β Ser9, β-catenin, EMT-related Wnt target genes)	A2780 and SK-OV-3 epithelial ovarian cancer lines	[116]
	Aloe-emodin	Inhibition (reduces Wnt3a, increases p-β-catenin/β-catenin ratio, reduces p-GSK3β)	Melanoma cell lines A375 and SK-MEL-28, in vivo model	[117]
		Inhibition (decreases β-catenin, cyclin D1, c-Myc, pro-oxidant effects)	Androgen-independent DU145 prostate cancer cells	[118]
	ethylamino-emodin	Inhibition (direct inhibitor of GSK3β, complex effects on β-catenin localization)	HepG2, HEK293, and primary hepatocytes	[119]
**2.12. N-containing compounds**	Ricinine	Activation (stimulates Wnt cascade, target is CK1)	HEK293 TopFlash reporter cells	[36]
	Berberine	Activation (promotes total and nuclear β-catenin)	Bone mesenchymal stem cells	[38]
		Inhibition (reduces cytoplasmic β-catenin)	HCT116 human colon carcinoma cells	[120]
	Matrine	Inhibition (inhibits VEGF, regulates Wnt/β-catenin signaling)	Breast cancer cells	[37]
		Inhibition (via PI3K/AKT/mTOR and AKT/GSK3/β-catenin pathways)	Hepatocellular carcinoma (HCC) cells	[121]

## Abbreviations

The following abbreviations are used in this manuscript:

NSCLC	Non-small-cell lung carcinoma
FZD	Frizzled
DMEM	Dulbecco’s modified eagle medium
TNBC	Triple-negative breast cancer
CCRK	Cell-cycle-related kinase
EMT	Epithelial–mesenchymal transition
MBP	Myelin-binding protein
ERα	Estrogen receptor α
HCC	Hepatocellular carcinoma
